# Biophysical Characterization of Cancer-Related Carbonic Anhydrase IX

**DOI:** 10.3390/ijms21155277

**Published:** 2020-07-25

**Authors:** Katarina Koruza, A. Briana Murray, Brian P. Mahon, Jesse B. Hopkins, Wolfgang Knecht, Robert McKenna, S. Zoë Fisher

**Affiliations:** 1 Department of Biology & Lund Protein Production Platform, Lund University, Sölvegatan 35, 22362 Lund, Sweden; katarinakoruza@gmail.com (K.K.); wolfgang.knecht@biol.lu.se (W.K.); 2 Department of Biochemistry and Molecular Biology, University of Florida, Gainesville, FL 32610, USA; murraya8@ufl.edu (A.B.M.); rmckenna@ufl.edu (R.M.); 3 Department of Molecular Biology, Princeton University, Princeton, NJ 08544, USA; bpmahon@princeton.edu; 4 The Biophysics Collaborative Access Team (BioCAT), Department of Biological Sciences, Illinois Institute of Technology, Chicago, IL 60616, USA; jhopkins1@iit.edu; 5 Scientific Activities Division, European Spallation Source ERIC, Tunavägen 24, 22100 Lund, Sweden

**Keywords:** carbonic anhydrase, small angle X-ray scattering, X-ray crystallography

## Abstract

Upregulation of carbonic anhydrase IX (CA IX) is associated with several aggressive forms of cancer and promotes metastasis. CA IX is normally constitutively expressed at low levels in selective tissues associated with the gastrointestinal tract, but is significantly upregulated upon hypoxia in cancer. CA IX is a multi-domain protein, consisting of a cytoplasmic region, a single-spanning transmembrane helix, an extracellular CA catalytic domain, and a proteoglycan-like (PG) domain. Considering the important role of CA IX in cancer progression and the presence of the unique PG domain, little information about the PG domain is known. Here, we report biophysical characterization studies to further our knowledge of CA IX. We report the 1.5 Å resolution crystal structure of the wild-type catalytic domain of CA IX as well as small angle X-ray scattering and mass spectrometry of the entire extracellular region. We used matrix-assisted laser desorption/ionization time-of-flight (MALDI-TOF) mass spectrometry to characterize the spontaneous degradation of the CA IX PG domain and confirm that it is only the CA IX catalytic domain that forms crystals. Small angle X-ray scattering analysis of the intact protein indicates that the PG domain is not randomly distributed and adopts a compact distribution of shapes in solution. The observed dynamics of the extracellular domain of CA IX could have physiological relevance, including observed cleavage and shedding of the PG domain.

## 1. Introduction

Carbonic anhydrases (CAs) are a family of metalloenzymes that catalyze the reversible hydration of CO_2_ in the presence of water, to HCO_3_^−^ and H^+^ [1]. The CA family consists of eight distinct classes, including the α-CA class, which is the only class expressed in humans [2]. Among the 12 catalytically active human α-CAs, only the membrane-bound CA IX and CA XII have been linked to cancer [3,4]. CA IX is gaining a lot of attention, as its expression is associated with more aggressive forms of cancer, its expression is upregulated by hypoxia, and has low expression in normal tissues [5]. CA XII is also cancer-associated, with different cancer-type distributions compared to CA IX, but its expression is not hypoxia-induced [6]. For example, in renal cell cancers that are defective in the Von Hippel–Lindau (VHL) gene, expression of CA IX is still high, even under normal oxygen conditions [7,8].

Within the tumor microenvironment, the hypoxia response upregulates glucose transporters, angiogenesis growth factors, and multiple enzymes of the glycolytic pathway through the transcription factor, hypoxia-inducible factor one alpha (HIF1α) [9,10,11]. Stabilization of the transcription factor HIF-1α leads to the expression of genes promoting tumorigenesis, angiogenesis, and the acquisition of a more glycolytic phenotype, rendering cancer cells independent of oxygen for ATP generation [12]. Increased glycolysis enhances the secretion of lactic acid to the extracellular space, via the monocarboxylate transporter MCT1 that is coupled to the co-transport of H^+^. This results in acidification of the extracellular space to maintain intracellular pH homeostasis [13,14,15]. In addition, the mitochondrial decarboxylation reactions contribute to extracellular acidification by generating CO_2_ that diffuses into the extracellular space, where it is hydrated to H^+^ and HCO_3_^−^ by CA IX, and in some cancers, also by membrane-bound CA XII [15,16].

Increased extracellular acidification promotes extracellular release and activity of proteases that break down extracellular matrix and promote migration, invasion, and metastasis [4,15,17,18]. Inhibiting the catalytic activity of CA IX appears to affect the pH maintenance of the tumor microenvironment, resulting in reduced tumor cell survival and proliferation [19,20]. All this makes CA IX an attractive target in cancer diagnosis, imaging, and treatment [21]. While CA XII expression is also associated with some cancer types, it is expressed more broadly across tissue types and expression is not regulated by hypoxia as it is for CA IX [6,22]. While the presence of CA XII has also been strongly associated with malignancy in certain cancers [23], its presence in breast cancer specifically is indicative of better patient outcomes [6]. However, in glioblastoma, it has been demonstrated that the inhibition of CA XII seems to reverse resistance to chemotherapy [24], making its role in cancer progression ambiguous.

In general, due to the high sequence and structural conservation between human CAs, there are significant challenges in CA drug administration. Due to off-target binding causing unwanted side-effects and reduction in drug efficacy [20,25], there is a need for isoform-specific inhibitors or binders that could be developed into clinical tools for cancer detection and treatment. 

**Figure 1:**
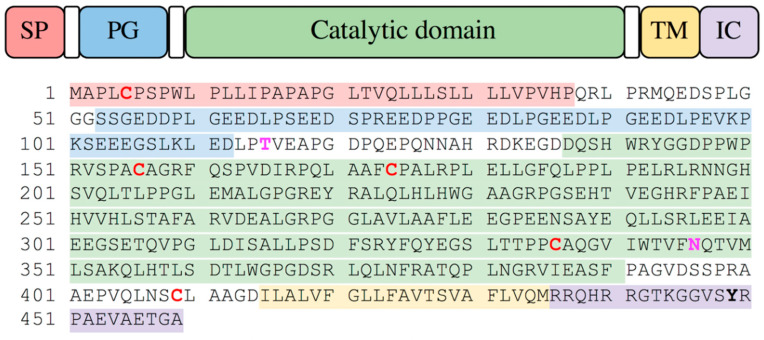
Sequence of native, full length human CA IX. The domains are shown in the image and mapped onto the sequence below using the same colors. Cys residues are shown in red and bold, glycosylated residues are shown in magenta, and phosphorylated residue in bold.

The CA IX numbering used in this paper refers to the full-length protein, including its signal peptide (SP) (Figure 1; Swiss-Prot entry Q16790). Human CA IX is a transmembrane protein, which consists of an N-terminal signal peptide (SP; residues 1–37), an extracellular domain (ECD; residues 38–414), a single transmembrane (TM) helix region (residues 415–435), and a cytoplasmic (CY) region (residues 436–459). The ECD consists of two conserved domains: The N-terminal proteoglycan-like (PG) domain (residues 38–112) and the CA catalytic domain (residues 139–390). There is a short stretch of amino acid residues connecting these domains (residues 113–138), while residues 391–414 are found between CA and the TM region.

Structural studies have shown CA IX forms an intramolecular disulfide bond (Cys156–Cys336) and a symmetric intermolecular disulfide bond (Cys174) involved in dimerization of the enzyme on the surface of the cell [26]. CA IX has a number of post-translational modifications that have been identified: O-linked glycosylation by heparan or chondroitin sulfate glycosaminoglycan chains at Thr115 in the N-terminal of the PG domain [26,27], N-linked glycosylation of high mannose sugar chain at Asn346 localized on the catalytic domain [26], and three phosphorylation sites on CY region, namely Thr443, Ser448, and Tyr449 [28,29]. The effect of these modifications on CA IX in pH regulation, cell adhesion, and tumor progression has not been fully understood, however, the perturbed glycosaminoglycan modification results in increased internalization and cytotoxicity of an antibody drug conjugate targeted to CA IX [27]. The overall fold and active site of CA IX is homologous to other α-CAs and has a mixed α/β-topology and a conserved zinc active site [21].

The net negatively charged PG domain was named due to its homology with a keratan sulfate attachment domain of a human large aggregating proteoglycan, aggrecan [30]. Notably, the PG domain is a unique feature of CA IX among human CAs and influences the catalytic efficiency of the enzyme. It has been shown that the catalytic activity of the entire extracellular domain (ECD), including the PG domain, was greater than that of the catalytic CA domain alone [26,31]. Previous studies suggested that the acidic residues in the PG domain could act as a proton buffer to facilitate CA activity at acidic extracellular pH in hypoxic cells [32]. Recent studies of CA IX have also shown that the PG domain could also act as a proton antenna and facilitate the movement of lactate and protons by monocarboxylate transporters in human breast adenocarcinoma cells [33].

Studies by Langella et al. showed that the PG domain belongs to the family of intrinsically disordered proteins (IDPs), being inherently highly flexible and mainly unfolded. There are local tendencies in the N-terminal region (residues 38–87) that assume polyproline II-like (PPII) conformations and may be involved in binding partner recognition [31,34]. The PG domain is reported to play a role in several cellular processes including cell–cell adhesion and intercellular communications [35,36]. The C-terminal region (residues 88–136) of the PG domain adopts a slightly more compact conformation that may have a role in modulating CA catalytic activity [31].

One feature of CA IX is that it is difficult to express a form of the ECD that forms well-ordered crystals, a pre-requisite for structure-based drug design. To date, only the catalytic domain of the enzyme has been structurally characterized using X-rays from a variety of different constructs and expression systems (PDB codes 3iai, 5dvx, 5fl4, 5fl5, and 5fl6) [20,21,37]. Attempts to crystallize the ECD of CA IX were first reported by Hilvo et al. and Alterio et al., but resulted in protein degradation that was assigned to protease contaminants within the purification/crystallization solutions [21,26]. These studies used four constructs, including the PG and CA IX domains. The diffraction data from ECD crystals were previously reported to 3.2 Å resolution, but there was no electron density observed for the PG domain. This was consistent with sodium dodecyl sulfate−polyacrylamide gel electrophoresis (SDS-PAGE) analysis of dissolved crystals, where a band migrating at the expected molecular weight of the CA IX catalytic domain alone was observed and this was further confirmed by matrix-assisted laser desorption/ionization time-of-flight (MALDI-TOF) peptide mass fingerprinting analysis [21]. An additional crystal structure was reported but not deposited in the PDB, despite being used as a starting model for determining the structure of the deposited model containing a Cys174Ser variant (PDB ID 3iai). In the work by Alterio et al., the recombinant protein was produced using the baculovirus expression vector system (BEVS). The His-tag was also removed prior to crystallization [21].

The construct used for the yeast-based expression of the catalytic domain of CA IX (residues 137–390; PDB ID 5fl4, 5fl5, and 5fl6) included the Cys174Ser substitution and the Asn346Gln substitution to avoid glycosylation, and was fused to maltose-binding protein (MBP) [7,20]. The protein was proteolytically cleaved and separated from MBP with affinity chromatography and crystallized in the presence of CA inhibitors [37].

The construct used for bacteria-based expression of catalytic domain of CA IX (residues 137–395; PDB ID 5dvx) included the Cys174Ser and five additional substitutions (Leu180Ser, Ala210Lys, Ala258Lys, Phe259Tyr, and Met360Ser) intended to improve crystal contacts and stability [20]. Except for 5dvx, all the constructs described above have an inhibitor bound (Acetazolamide and 2-thiophene-sulfonamide) that were reported to improve enzyme stability during crystallization [20,21,37].

Here, we report an integrated structural biology approach to characterize the CA IX ECD expressed in *Spodoptera frugiperda* (Sf9) cells using the Baculovirus Expression Vector System (BEVS). BEVS was chosen as an expression system to maximize the chances for producing glycosylated and soluble CA IX ECD. A combination of MALDI-TOF mass spectrometry (MS), small angle X-ray scattering (SAXS), and X-ray crystallography has been used to further our knowledge of the ECD of CA IX. The constructs were coded for amino acids 1–414 (Uniprot ID Q16790) corresponding to the signaling peptide, PG domain, and the catalytic CA domain—similar to previous descriptions [21,26,38].

## 2. Results and Discussion

During our efforts to study the crystal structure of the entire ECD of CA IX, we observed spontaneous degradation of the protein by SDS-PAGE and visible precipitation of the purified protein. Surprisingly, this degradation enabled crystallization of the soluble and stable catalytic domain of CA IX, while the remaining insoluble product was further characterized with mass spectrometry. The crystal structure of the CA domain we report is for wild-type CA IX (without amino acid substitutions) to 1.53 Å resolution and we were able to resolve residues Asp137–Val394. There is excellent density for the active site residues and solvent. Small angle X-ray scattering (SAXS) analysis of the intact protein indicated that the PG is inherently disordered but is loosely compact and occupies a preferred position.

### 2.1. Production of Recombinant Protein, SDS-PAGE and Degradation

Soluble recombinant Constructs 1 and 2 were produced using the baculovirus expression vector system (BEVS). For crystallization and mass spectrometry of Construct 1, the 8x-His-tag was left intact and the fresh protein appeared soluble and initially quite stable at 4 °C.

**Figure 2:**
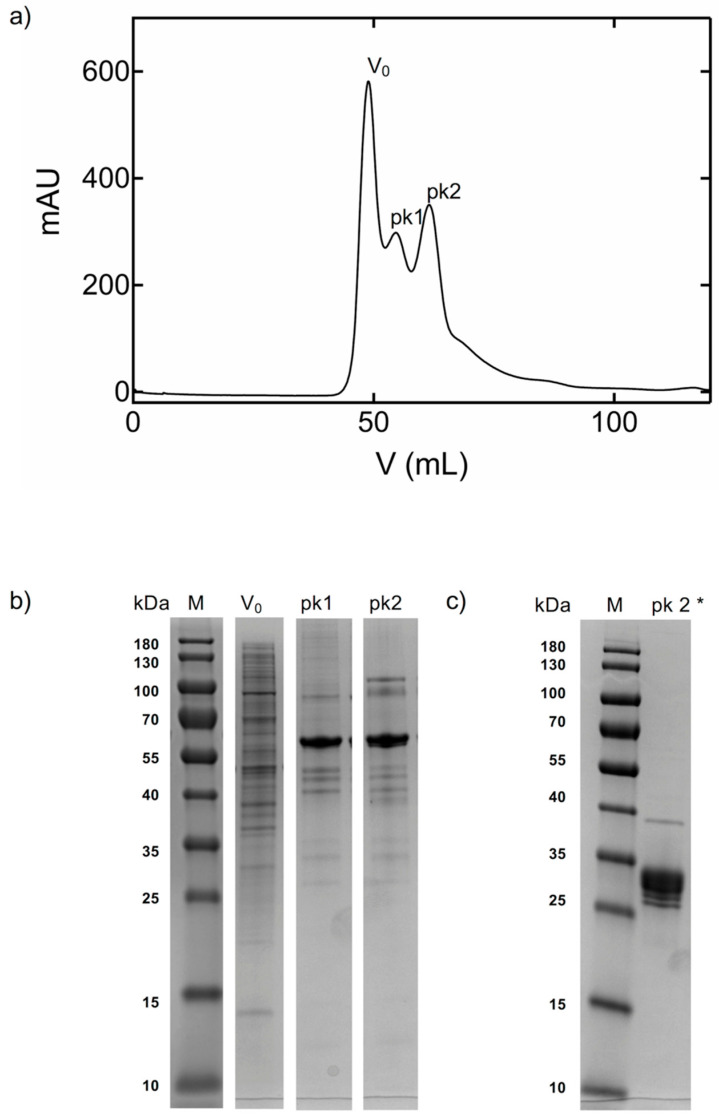
Chromatograms and SDS-PAGE from size exclusion chromatography peaks (Superdex 200 16/600, GE Healthcare, Chicago, IL). (**a**) CA IX elutes after the expected void volume (V_0_) as peak 1 (pk1) and peak 2 (pk2). X-axis indicates fractions and volume; Y-axis is absorption units; (**b**) SDS-PAGE (18%) stained with Coomassie Brilliant Blue. Molecular masses (M) of broad range protein marker (10–180 kDa) (Thermo Scientific) are indicated in kDa. Analysis of peak fractions corresponding to peak 1 and peak 2. (**c**) “degraded” peak 2 (marked as pk 2 *) after 6 months storage at 4 °C.

As reported by others, oligomerization of CA IX was also observed under native conditions in size-exclusion chromatography (SEC) [26]. The SEC chromatogram of Construct 1 CA IX showed two major peaks (Figure 2) that were poorly resolved due to the use of a low-resolution preparative grade gel filtration column. The elution positions of peak 1 and peak 2 could correspond to dimeric and monomeric ensembles of CA IX as they contain similarly sized proteins when analyzed on SDS-PAGE under denaturing conditions. It should be noted here that the monomeric protein migrates at a higher than theoretical calculated molecular weight (~47 kDa) on SDS-PAGE. This could be due to a number of reasons, including glycosylation of residue Thr115 found between the PG and CA catalytic domain, and Asn346 located in the CA IX catalytic domain (Figure 1, bold).

Only peak 2 (monomer) of Construct 1 was used for successful crystallization in this study and it was observed that after several months at 4 °C, the protein spontaneously degraded, leaving behind a stable CA IX catalytic domain in the soluble portion (Figure S1a). Inspection of the soluble protein on SDS-PAGE gels reveal a band migrating at a MW corresponding to the monomeric CA catalytic domain only (~28 kDa) (Figure 2). There are multiple closely clustered bands on the gel around 28 kDa, suggesting alternate N- or C-termini or variation in post-translational processing. As discussed later, neither mass spectrometry nor crystallography were able to help resolve the exact start and/or end residues of the crystallized protein after degradation/cleavage.

The absence of the entire PG domain was due to quick degradation and could be due to the presence of low levels of protease contaminants. The cleavage was fortuitous, as only the degraded protein crystallized and yielded very well-diffracting crystals (Table 1). Crystallization experiments were unsuccessful with fresh protein made from Construct 1 and took weeks to a month to obtain crystals from proteolyzed protein, suggesting that the PG domain interferes with ordered protein packing to produce a crystal.

### 2.2. MALDI-TOF Mass Spectrometry

**Figure 3:**
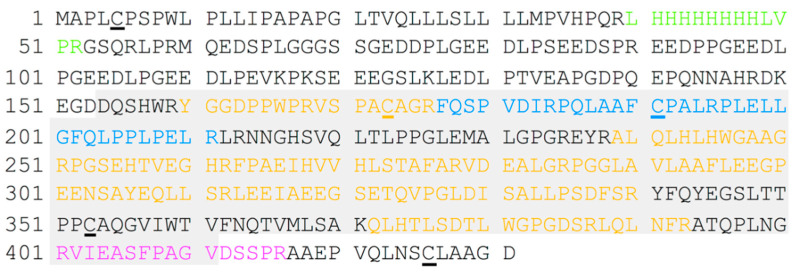
Sequence of Construct 1 used for crystallography and mass spectrometry. Shaded in gray is the extent of the CA domain visible in the crystal structure, while peptides from different samples are shown in different colors as follows: blue are the peptides only seen in the crystal; magenta is for the soluble portion; yellow are the peptides seen in both the soluble sample and the crystal; green is for the peptide containing the N-terminal His-tag that was only seen in the insoluble (precipitated) sample. Cys residues in the expression sequence are underlined.

In order to understand the state of the protein (Construct 1) that crystallized and possibly determine the new N- and C-termini after cleavage, we analyzed three different samples with MALDI-TOF MS (Figure 3). Both intact protein and trypsin digested protein (peptides) were measured. The samples submitted were as follows: (a) soluble supernatant from sample at 4 °C, (b) insoluble precipitation from (a), and (c) washed and crushed crystals from same plate that the crystal structure was determined from. 

Broad and noisy peaks for the intact measurements for (a) and (b) made it difficult to obtain an accurate mass (Figure S1c,d in the Supporting Material). Estimation of mass using full-width at half the maximal height of the peaks gives ~28 kDa, consistent with what is calculated from the crystal structure and migration on SDS-PAGE (Figure 2).

Tryptic digest and MALDI-TOF MS results of the peptides are shown in Figure 3. Identified peptides are shown in different colors and overlaid with the expression construct sequence. The extent of the sequence resolved in the crystal structure is shown in gray. The soluble and crystallized samples (Figure S1(a1,a3)) share many of the same peptides, with one additional peptide that was observed in the crystal sample only (Figure 3, Figure S1b). All the peptides found in the soluble fraction and the crystal were within the sequence of the crystal structure and provide good coverage of the CA catalytic domain. The MS results did not unambiguously reveal the identity of the N- or C-termini of the protein in the crystal. The insoluble sample shared many of the same peptides from the soluble and crystalline samples, but also had an additional peptide from the N-terminal region of the protein that included the His-tag. This suggests that the PG domain is only present in the insoluble portion and not in the soluble sample or the crystal (Figure 3, Figure S1). 

The intact measurements of the insoluble portion in the reflector mode revealed the presence of peptides with a strong peak at ~1960 Da. This was not due to cleavage by trypsin and further investigation in Expasy Peptide Cutter (https://web.expasy.org/peptide_cutter/) revealed that neutrophil elastase could cleave the protein to yield a peptide of this mass (sequence: HPQRLHHHHHHHHLV, Figure S2) [39]. It has been shown that Thr115 is O-glycosylated and this could explain peptide heterogeneity in the insoluble portion of the expressed protein [26]. The MS results also support what was observed in solution and on SDS-PAGE: the expressed protein containing the PG domain is unstable and becomes insoluble over time. The residual protein, after cleavage of the N-terminal portion containing the His-tag and PG domain, is the CA IX catalytic domain and seems stable and soluble as it is amenable to being concentrated and crystallized. 

### 2.3. Small-Angle X-ray Scattering (SAXS)

**Figure 4:**
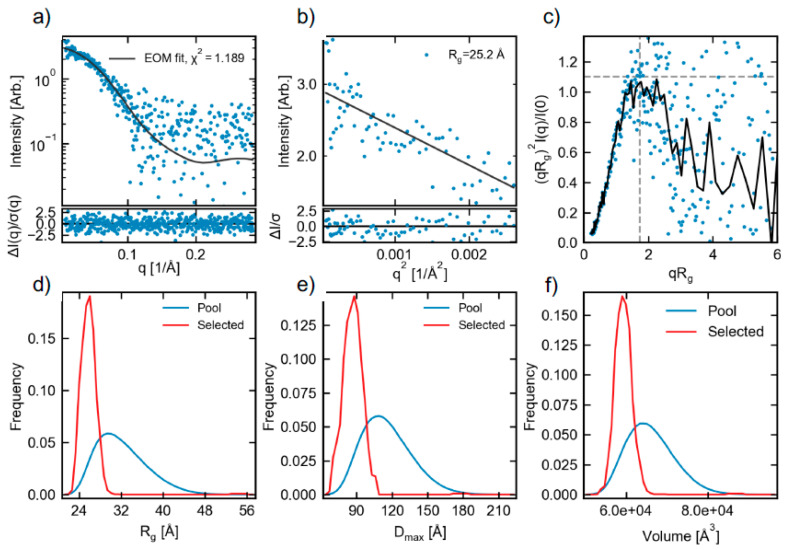
(**a**) SEC-SAXS scattering profile (blue points), EOM fit to the data (black line), and the normalized fit residual (bottom panel). (**b**) Guinier region for data (blue dots), Guinier fit (black line), and normalized fit residual (bottom panel). (**c**) Dimensionless Kratky plot. The dashed gray lines show where their peak for a compact globular protein would be. The black line is a log-rebinned version of the data, and fails to fall to 0 around qRg of ~5, which is what would be expected from a globular system. This suggests the measured system is flexible. (**d**–**f**) EOM results for the R_g_, D_max_, and volume, respectively, of the total pool (blue) and selected ensemble (red) of flexible structures in solution. In all three cases, the selected ensemble is much more compact than the possible pool of structures, which shows that the PG domain does not adopt all possible states in solution, but rather, is constrained to states mostly near the CA IX domain.

**Figure 5:**
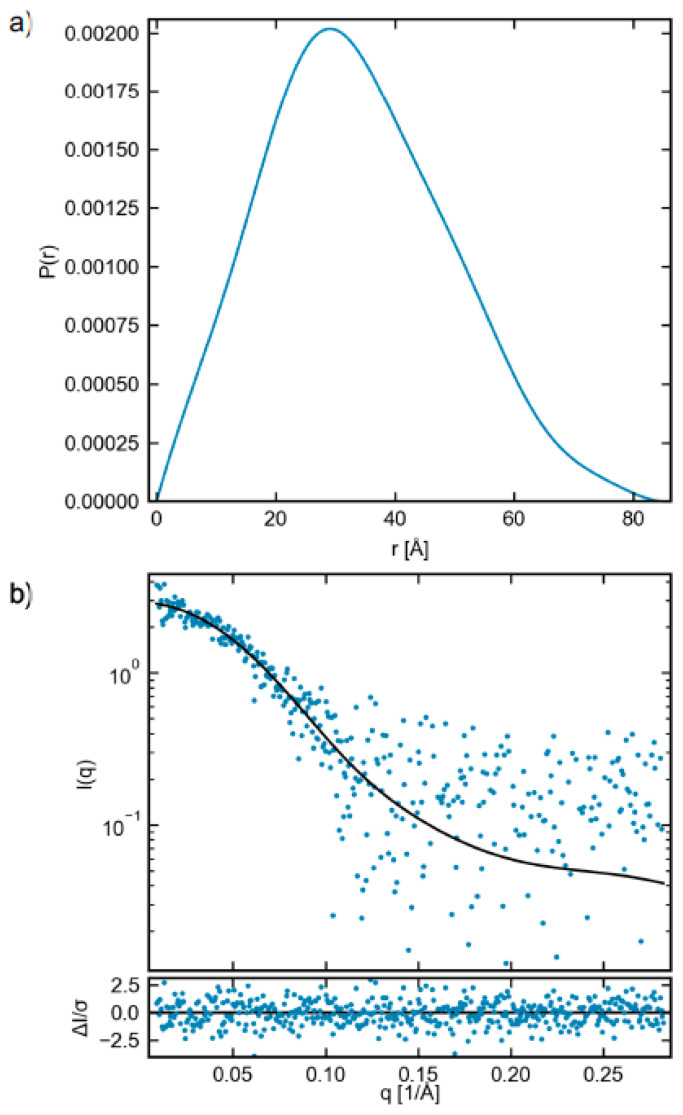
(**a**) P(r) function (blue line) as calculated from the SAXS data, normalized by I(0). (**b**) The scattering profile (blue points), fit to the data from the P(r) function (black line), and normalized fit residual (bottom panel).

The scattering profile obtained via SEC-SAXS for freshly prepared protein from Construct 2 is shown in Figure 4a. A Guinier fit, Figure 4b, gave the radius of gyration (R_g_) of 25.2 ± 0.9 Å. The molecular weight calculated from the Guinier fit via the volume of correlation, the most appropriate method of flexible systems [40], was 36.1 kDa. While a slight underestimate of the expected MW, this method depends strongly on the high q data. The low signal-to-noise of the profile at high q likely leads to this underestimate, and can be shown by truncating the scattering profile to q = 0.25 (from 0.28), giving a MW of 41.1 kDa very close to the expected value for the construct of 40.9 kDa (calculated from sequence). This gives confidence that the measured system is the ECD of CA IX with the PG domain. A normalized Kratky plot, Figure 4c, can show evidence of flexibility. A highly binned version of the data (black curve) does show a longer tail characteristic of flexibility, as the normalized Kratky plot for a globular system would fall to zero around qRg of ~5 [41]. Given the noise level of the data at high q, this is suggestive but not definitive evidence of flexibility. The P(r) function, Figure 5, gave a maximum dimension (D_max_) of 85 Å. The shape of the P(r) function, with an asymmetric peak at lower distance and an extended tail, is characteristic of a system with both folded and flexible regions [42,43]. A purely globular system would have a more symmetric P(r) function, with the peak shifted to a higher distance [44].

Bead model reconstructions of the data were done using DAMMIF and DAMMIN. Averaging with DAMAVER gave a normalized spatial discrepancy (NSD) of 0.995, with one model rejected from the average. Clustering with DAMCLUST showed two non-isolated clusters. The ambiguity assessment of the scattering profile via AMBIMETER, indicating how ambiguous bead model reconstructions may be, had an ambiguity score of 2.24, interpreted by AMBIMETER as “3D reconstruction might be ambiguous”. This high NSD, ambiguity, and the existence of several clusters are all characteristic of flexible systems. Because of the poor quality of the reconstructions, the models are not shown.

As previously described, the PG domain is expected to be highly flexible. The evidence from the SAXS analysis described above supports at least some degree of flexibility in the system. In order to model the flexibility, EOM was used. This generates a pool of possible structures based on assigned flexibility to the system. A genetic algorithm then selects a subset of the total pool that fits the data. Comparing the size distribution (R_g_, D_max_, and volume) of the selected ensemble to that of the full pool can provide quantitative information on the degree of flexibility in the system. The R_g_, D_max_, and apparent volume of the generated pool and the selected ensemble are shown in Figure 4d–f. The fit of the selected ensemble to the data is shown in Figure 4a. The selected ensemble is clearly much less extended than the full pool, and shows much less spread in the size of the molecule. This shows that while the system is flexible, it is significantly less flexible and less extended than you would expect were the PG domain in fully random configurations in solution. In particular, the PG domain mostly exists in conformations that remain close to the CA IX domain, rather than ones extended out into solution.

The statistics of the full pool and the selected ensemble also show the more compact and less flexible nature of the molecule, relative to the possible states. For the R_g_, the full pool average is 32.1 Å, while the selected ensemble average is 25.9 Å. The average D_max_ of the full pool is 115.6 Å, while for the selected ensemble, it is 87.0 Å. The average apparent volume for the full pool is 66,000 Å^3^, while the average selected ensemble average volume is 59,400 Å^3^. EOM also provides more advanced metrics for assessing flexibility. R_flex_ describes the entropy of the system, on a scale from 0 (rigid) to 100% (completely disordered); a more flexible system has a larger R_flex_. The R_flex_ value from the selected ensemble was 52.7%, while for the pool, it was 83.3%. The EOM program also provided an interpretation of the solution conformations as “very compact”, which agrees with our assessment of the results.

EOM also provides the R_sigma_ metric, which describes the spread of values in the ensemble relative to the pool. Normally, for a compact state, this would be less than 1. For this dataset, it is 1.66. This is due to a small amount of extremely extended states in solution, most visible in the very small peak in the selected ensemble D_max_ distribution near 180 Å, Figure 4e. This shows that while the majority of the time the system is in a very compact state, there is a small fraction of states that are highly extended in solution.

**Figure 6:**
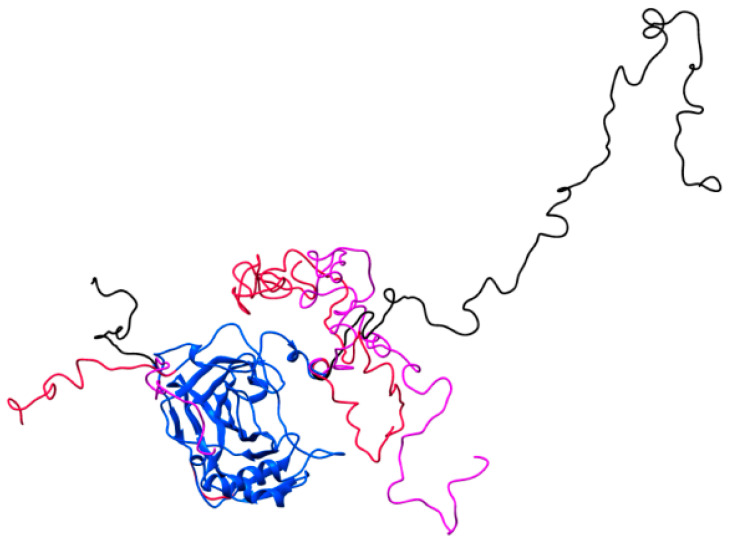
The best fit ensemble models of CA IX and PG domains from EOM analysis of SEC-SAXS data. Here, the core catalytic domain (shown in blue) CA IX from the crystal structure (PDB ID 3iai) was kept fixed, and EOM determined the possible conformations of the PG domain, which are highlighted in a range of colors and as random-loops. Compact structures representative of ~90% of the states in solution are shown in red and purple, while an extended structure, representative of the remaining minority conformations in solution, is shown in black. These models are representative of common structures in solution, but are meant only to aid in interpretation of what compact states are, and do not show the full range of possible solution structures.

Overall, the SAXS results support the conclusion that the system is highly flexible, but show that the PG domain is not randomly distributed and adopts a compact distribution of shapes in solution. This is illustrated in Figure 6, where three representative structures from the best fit ensemble aligned on the catalytic domain are shown. Two, shown in red and purple, representing ~90% of the selected ensemble, show compact structures, while the other shown in black is an extended structure.

### 2.4. Crystallography

The soluble portion of the sample derived from Construct 1 was used to set up crystallization trays and yielded well-diffracting crystals to 1.5 Å resolution (crystals shown in Figure S1(a3) in the Supporting Material). Data collection and model refinement statistics can be seen in Table 1. The space group was determined to be C2 with unit cell dimensions: *a* = 113.9, *b* = 78.3, *c* = 74.3 Å, β = 128.2°. This corresponds to a unit cell volume of ~518,000 Å^3^ and contains two chains in the asymmetric unit cell. Several hydrophilic and hydrophobic residues mediate the interactions to form the non-crystallographic dimer with a buried surface area of ~1360 Å^2^. Interface interactions are listed in Table S2. The interactions between the two chains in the asymmetric unit cell do not include disulfide bond formation by Cys174 and we conclude that this dimer is probably not physiological, but instead, a crystallographic packing event, induced during the removal of solvent as the protein concentration is increased.

The two chains are superimposable with a least-squares fit of chain A onto B of 0.35 Å for all atoms. The following rotation and translation parameters can be used to generate the position of the second chain: rotation (polar: omega, phi, kappa in degrees): 128.08, 179.27, 179.66; Translation (x, y, z in Å): −46.7, 34.7, 60.5.

Analysis of the 2F_o_-F_c_ and omit F_o_-F_c_ electron density maps allowed building and placement of the core CA catalytic domain with no additional density corresponding to PG domain or C-terminal residues present in the expression construct (Figure 3).

This is consistent with what was observed on SDS-PAGE and with MALDI-TOF MS of the crystals. The N-linked glycosylation on Asn354 was clearly visible for three saccharide units (Figure S3 in the Supporting Material). Overlay of the current structure A chain onto the A chain of the deposited 3iai structure had an RMSD of 0.45 Å for all atoms and visual inspection indicated the structures were effectively the same.

**Figure 7:**
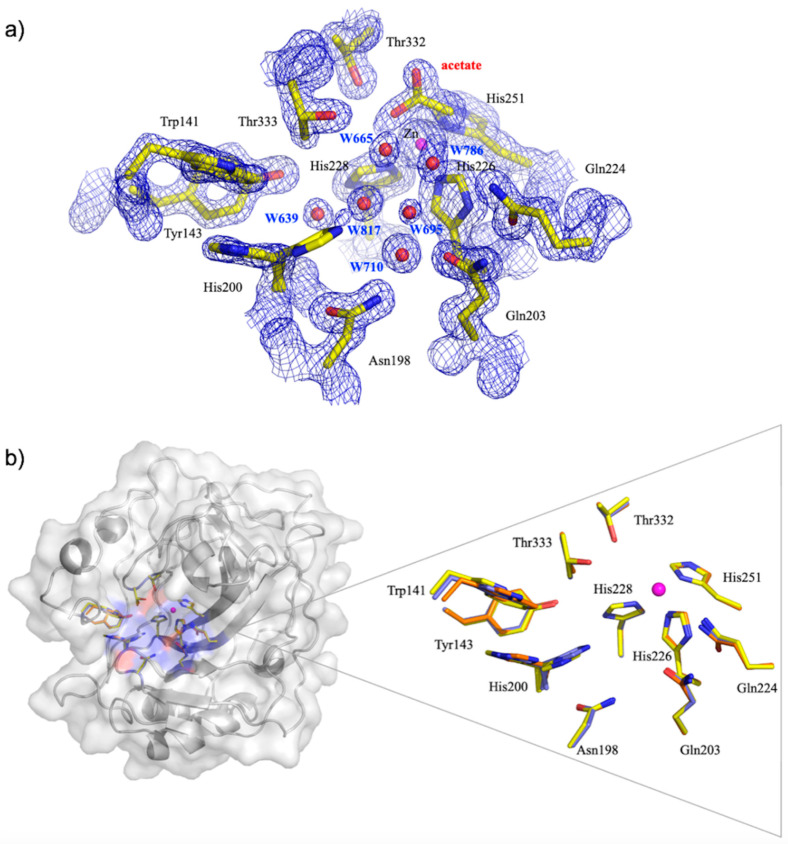
(**a**) Active site of CA IX in complex with acetate shown as sticks. 2F_o_–F_c_ electron density map is shown in blue mesh with 1.5σ level for the protein/solvent/ligands and 3.0σ for the Zn. Residues, solvent, and ligands are as labeled. (**b**) Overlay of active sites of current CA IX structure (blue) with CA IX surface variant (yellow) from PDB ID 6rqn and CA IX from PDB ID 3iai (orange) [21,46].

The active site is situated at the base of the conical cleft lined on the one side with hydrophilic amino acid residues (shown in Figure 7a,b: Tyr143, Asn198, His200, Gln203, Gln224, Thr332, Thr333). The hydrophilic residues coordinate an ordered network of solvent molecules that connect the Zn^2+^ ion to the bulk solvent via His200 (Figure 7a). In the structure reported, we observed density that could only be modeled as an acetate molecule adjacent to the Zn^2+^ ion and Thr332. Small anions such as sulfate and acetate have some affinity for the active site of CA and despite not having acetate in the crystallization condition, we do observe density corresponding to acetate in the structure (Figure 7a) [45]. Previous structures of native CA IX contained inhibitors (e.g., Acetazolamide in PDB 3iai) as well, but as acetate is a small molecule, we are still able to observe several well-ordered water molecules in the active site of CA IX and the proton shuttle, His200 (His64 in CA II), is also in both in the “in” and “out” conformations.

Figure 7b shows an overlay of the amino acid residue sidechains and Zn^2+^ ion from the current structure (in blue), 3iai (orange), and CA IX surface variant 6rqn (yellow). The residue positions and conformations are homologous between these three structures, indicating that the size and nature of the bound inhibitor does not have a disruptive effect. The only residue that is slightly different is His200. In the 3iai structure with acetazolamide bound, His200 is all in the “out”, while in the other two structures it is both “in” and “out”.

The MALDI-TOF MS did not give a clear indication where the N- and C-termini were for this protein after spontaneous cleavage. Both were also poorly ordered in the crystal structure and we were able to build from Asp137 to Val406 (269 amino acids in total). In the part that crystallized, there are three Cys residues with very good density (Figure 3). Cys156 and Cys336 are engaged in an intramolecular disulfide bond, while Cys174 does not interact with any other molecules/residues and is observed in two conformations and facing a solvent channel in the crystal. This means that in our crystal structure, Cys174 is not involved in an intermolecular disulfide bond to generate the observed non-crystallographic dimer. Whether the dimer observed in this crystal structure is the same as the dimer observed in the SEC is unknown.

## 3. Materials and Methods

### 3.1. Expression and Purification of ECD of CA IX 

Production of the ECD of CA IX (PG domain + catalytic domain) using the BEVS was modified after the methods described previously [21,26,38]. We used 2 constructs, described in detail below. The amino acid residues included in the constructs (UniProt Q16790) included the signal peptide, PG domain, and the CA catalytic domain (residues 1–414). They are identical except for inclusion of Cys174Ser in Construct 2. Both had an 8xHis-tag and Thrombin-cleavage site inserted between the signal peptide and PG domain, as previously described [21,26,38]. 

The wild-type cDNA construct (referred to here as Construct 1) was synthesized by GeneArt (ThermoFisher, Waltham, MA, USA) and subcloned into pVL1393 (BD Bioscience, Franklin Lakes, NJ, USA) [47]. Construct 2 containing the Cys174Ser mutation was a generous gift from Claudiu T. Supuran (University of Florence, Italy) and Seppo Parkkila (University of Tampere, Finland). During the purification of Construct 1, the His-tag was retained, while for Construct 2, the His-tag cleaved off. Below, follow the methods used for expression and purification of both constructs.

### 3.2. Construct 1 Protein Expression and Purification

Details of protein production for Construct 1 has been described previously [47]. Briefly, the protein was produced using BEVS and expression was done in Sf9 cells (Invitrogen, Carlsbad, CA, USA). Purification was done by immobilized metal affinity chromatography (IMAC) using HisTrap Excel (GE Healthcare, Chicago, IL, USA) columns equilibrated in buffer (50 mM sodium phosphate, 500 mM NaCl, pH 8.0). Bound protein was eluted by increasing concentrations of imidazole up to 500 mM in the same buffer. A final purification step was done by size exclusion chromatography (SEC) using Superdex200 column (GE Healthcare, Chicago, IL, USA) in 50 mM Tris/HCl, 150 mM NaCl, pH 8.5. The protein also eluted in two peaks (peak 1 and 2) after a large peak at the expected void volume (V_0_). The fractions were analyzed by sodium dodecyl sulfate-polyacrylamide gel electrophoresis (SDS-PAGE) to assess protein purity and homogeneity (Figure 2).

Eluted fractions from peak 1 and 2 containing target protein were separately pooled and concentrated to 10–12.6 mg/mL, by Amicon Ultra Centrifugal Filter Units (Merck, Darmstadt, Germany) with molecular weight cut-off 10 kDa. Protein from peak 1 and peak 2 was stored at 4 °C and used within 24 h of purification to set up initial crystallization plates. The remaining protein was then stored for approximately 6 months at 4 °C. During this time, some of the soluble protein precipitated and was observed as a white non-soluble aggregate at the bottom of the tube. The soluble part left in the tubes from both peaks was once again assessed for protein purity and homogeneity by SDS-PAGE analysis (Figure 2) and also used to set up crystallization plates.

### 3.3. Construct 2 Protein Expression and Purification

The baculovirus for Construct 2 was done using the Bac-To-Bac^TM^ system (Invitrogen, Carlsbad, CA, USA). Sf9 cells (1 L) were grown in Sf-900 II SFM medium (Gibco, Waltham, MA, USA) to a density of 2.0 × 10^6^ cells mL^−1^ and were infected at a MOI of 5. Cells were harvest at 3 days post infection and the supernatant containing Construct 2 was diluted 2-fold in 20 mM sodium phosphate, 500 mM NaCl, 15 mM imidazole, pH 7.8.

Protein purification was done in a multistep process. The first purification step used was IMAC using the Probond Purification System (ThermoFisher, Waltham, MA) equilibrated in binding buffer (25 mM sodium phosphate, 250 mM NaCl, 10 mM imidazole, pH 8.0). Bound protein was eluted with 50 mM sodium phosphate, 500 mM NaCl, 250 mM imidazole, pH 8.0 [26]. Construct 2 was subsequently concentrated with centrifugal concentration devices to a volume of 500 μL before the poly-(8x)-His-tag was removed using the Thrombin CleanCleave Kit (Sigma-Aldrich, St. Louis, MO, USA) according to the manufacturer’s instructions. The sample was then further purified, and buffer-exchange was done through SEC with a Superdex 200 10/300 GL gel-filtration column (GE Healthcare, Chicago, IL, USA) equilibrated in 50 mM Tris/HCl, 150 mM NaCl, pH 7.4. Two individual peaks were observed (absorbance 280 nm), indicative of monomeric and dimeric protein populations. Fractions from both peaks were pooled and concentrated via centrifugation. The final concentration of purified Construct 2 was determined to be 3 mg/mL by UV–vis spectroscopy [48] using an extinction coefficient of 5.2 × 10^4^ M^−1^·cm^−1^.

### 3.4. MALDI-TOF Mass Spectrometry of Construct 1 

Different soluble and insoluble samples of CA IX construct 1 (Figure 3; Figure S1) were digested in solution with trypsin (2 μg trypsin:protein) in 50 mM Ammonium bicarbonate buffer, pH 7.8, at 37 °C overnight. MS spectra of the protein samples and the tryptic digested samples were acquired using an Autoflex Speed MALDI-TOF/TOF mass spectrometer (Bruker Daltonics, Bremen, Germany) in positive reflector (for peptides) or linear (proteins) mode. Matrix solution, 0.5 μL consisting of 5 mg/mL α-cyano-4-hydroxy cinnamic acid, 80% acetonitrile, 0.1% TFA, was added to 1 μL peptide or protein sample on a MALDI stainless steel plate. Peptide spectra were externally calibrated using Peptide Calibration Standard II (containing 9 peptides) from Bruker Daltonics and protein spectra were externally calibrated using intact BSA (containing 3 protein signals) from Bruker Daltonics. Raw files were converted to mgf-format files by Mascot Distiller (version 2.6, Matrix Science, Boston, MA, USA) and identification of proteins was carried out with the Mascot Daemon software (version 2.4, Matrix Science, Boston, MA, USA). The following search settings were used: trypsin as protease, 1 allowed missed cleavage sites, 40 ppm MS accuracy for peptides variable modifications: Oxidation (M). The result files were searched against an in-house created database and to be considered a hit, the peptide/protein had to score >100 using the full width at half maximum parameter.

### 3.5. Crystallization of Construct 1

Fractions corresponding to peak 1 and 2 from SEC (Figure 2) were concentrated and used to set up crystallization screens with fresh protein and with protein that was left for ~6 months. Three crystallization screens (JCSG+ (Molecular Dimensions, Newmarket, UK), PurePEGs (Anatrace, Maumee, OH, USA), and TOP96 (Anatrace, Maumee, OH, USA)) were set up using by the Oryx8 Automatic Protein Crystallization Systems (Douglas instruments, Westfield Farm, Hubbardston, UK) and 96-well plates, and incubated at 293 K. The protein:precipitant ratio used for sitting drop vapor diffusion set-ups was 1:1 (final drop volume 4 µL). It was observed that no crystals appeared when crystallization drops were made with fresh protein. The only crystals obtained came from the peak 2 sample that was left for ~6 months. The first crystals of proteolyzed protein were observed ~3 weeks after drop set-up in vapor diffusion format and came from the following condition: 20% PEG 6000, 100 mM Sodium citrate, 1 M LiCl, pH 4.0. These first drops were used to prepare seed stock according to the Seed Bead Kit instructions from Hampton Research. The seeds were then used for optimization of conditions and fine-screening using the Oryx8. The ratio used for microseeding experiments was protein:precipitant:seed of 1.5:1.0:0.5 (final drop volume 3 µL) and crystals appeared ~6 days after drop set-up.

The optimized crystallization condition was: 17% PEG 6000, 1.4 M Sodium citrate, 1 M LiCl, pH 4.3. This condition was also used for setting up larger volume drops in vapor diffusion (final drop volume up to 18 µL). All crystallization plates were incubated at 20 °C and inspected under a polarizing light microscope, or with the Minstrel HT UV imaging system (Rigaku, Tokyo, Japan). When harvested prior to X-ray data collection, crystals were cryopreserved by quick-dipping them into 20% glycerol solution prepared in 17% PEG 6000, 100 mM Sodium citrate, 1 M LiCl, pH 4.3, and stored under liquid nitrogen temperature until the X-ray diffraction data collection.

### 3.6. Crystallographic Data Collection and Structure Refinement

Data of Construct 1 were collected at 100 K on the BioMAX beamline at MAX IV laboratory (Lund, Sweden). Data processing was initially performed using the *autoPROC* software package [49]. The automated workflow script uses mainly XDS [50] as the data processing and scaling software, and POINTLESS for space group determination. The phases for the X-ray data were obtained by molecular replacement in Phaser [51] using PDB ID 3iai as a model [21]. The starting model was modified by removing all metals, ligands, and waters prior to searching. The X-ray structures were rigid-body refined in Phaser followed by restrained refinement in the PHENIX program suite [52]. For all datasets, a bulk solvent correction and a free *R*-factor monitor (calculated with 5% of randomly chosen reflections) were applied throughout the refinement. The map interpretation and manual model building was performed using Coot [53]. Figures were generated using *PyMOL* (http://www.pymol.org). The model coordinates and experimental data were deposited in the RCSB Protein Data Bank with the following accession code 6y74. Data collection and refinement statistics are summarized in Table 1. Dimer interface analysis, buried surface area calculation, and mapping of interactions were done in PyMOL, Coot, and using the PDBePISA server and are shown in Table S2 in the Supporting Material [53,54,55]. 

### 3.7. Small Angle X-ray Scattering (SAXS) of Construct 2

SAXS measurements were collected on freshly prepared and purified Construct 2 at the Cornell High-Energy Synchrotron Source (CHESS) BioSAXS beamline G1 (Now ID7A1; MacCHESS), operating at 9.96 keV (1.245 Å) and a sample to detector distance of 1498.2 mm [56]. Scattered intensity was recorded on a Pilatus 100 K detector, giving access to a q (q *=* 4*πsin θ/λ*) range of 0.0087–0.283 Å^−1^. SAXS was performed using in-line size exclusion chromatography (SEC-SAXS) to prepare homogenous and monodisperse protein for SAXS [57]. The sample was loaded onto a Superdex 200 5/150 column, run at 0.3 mL/min by an AKTA Purifier FPLC (GE Healthcare, Chicago, IL, USA) using 50 mM Tris/HCl, 150 mM NaCl, pH 7.5 as the mobile phase buffer. After the eluate passed through the UV monitor, it was flown continuously through the SAXS cell. The 1 s exposures were acquired continuously during exposure time, and data were reduced to 1D profiles using BioXTAS RAW version 0.99.14b. Reduced data were processed using BioXTAS RAW version 1.6.3 [58,59]. In-line SEC-SAXS showed two strong peaks (Figure S4 in the Supporting Material). Buffer subtraction was done using pre-peak buffer, and an average scattering profile was created from the data corresponding to the first peak. No useable SAXS data were obtained from the second peak, due to the small molecular size and low concentration. Guinier analysis was carried out in BioXTAS RAW, and molecular weight was determined in the same program by both the adjusted Porod volume and volume of correlation methods [40,60].

A P(r) function was generated using GNOM [61] from the ATSAS package. Ambiguity of bead model reconstructions was assessed by AMBIMETER [62]. Bead model reconstructions were carried out by running 15 reconstructions with DAMMIF [63] in slow mode, averaging and clustering the results with DAMAVER [64] and DAMCLUST [65], and then, refining the averaged output with DAMMIN [66].

Flexibility analysis was done using the ensemble optimization method (EOM) 2.0 [67]. The monomeric CA IX catalytic domain taken from a known crystal structure (PDB ID 3iai) was used as input to EOM, with the first 14 residues removed to allow for likely flexibility. EOM was used to generate 50,000 possible structures for the full pool using default settings and native-like structures. The EOM program fixes the position of all residues present in the input PDB and generates flexible structural elements whenever residues in the input sequence file are not present in the input PDB. The fixed regions of the pool structures are, thus, the defined positions within the input CA IX catalytic domain PDB file. The flexible regions that varied from structure to structure include the trimmed residues in the N terminus, several small undefined regions in the PDB (such as residues 233–236), and the entire PG domain. A sub-ensemble that matches the scattering data is, then, selected by a genetic algorithm. The genetic algorithm was run 10 times using default settings to verify the stability of the results (results from 1 run shown). All programs from the ATSAS package were from ATSAS version 2.8.4 [68]. Results of the SAXS analysis are presented in the supplemental Table S1 in accordance with the revised guidelines for publishing SAXS data [69]. The SAXS data are deposited in the SASBDB (https://www.sasbdb.org/) with access code SASDHT5.

## 4. Conclusions

Here, we reported the characterization of both the PG and the catalytic domain of the ECD of CA IX with mass spectrometry, SAXS, and crystallography. Our results indicate that the N-terminal portion including the PG domain is unstable and degrades over a few days and precipitates in vitro. The soluble portion that remained contained only the CA catalytic domain, then, crystallized readily, and we were able to determine a high-resolution crystal structure to 1.5 Å resolution. While no CA inhibitor was incorporated into the crystallization protocol, the active site Zn does have what appears to be acetate bound. The CA IX structure is a non-crystallographic dimer in space group C2 with a smaller unit cell compared to 3iai [21]. Seen also in the current structure is a different dimer interface mediated by hydrogen bonds and not involving a Cys residue, supporting the notion this is not a physiological dimer. This is despite the fact that it does not contain the Cys174Ser substitution, which would permit the proposed physiological dimer to form [21].

Until very recently, only limited information about the PG domain was available, despite the important role of CA IX in cancer and the uniqueness of this domain among human CAs. A role of the PG domain has been postulated for regulation of CA IX enzymatic activity, as it was measured that the catalytic activity of the entire ECD, including the PG domain, is greater than that of the catalytic CA domain by itself [26,31]. A potential role in cell adhesion and intracellular communication can be derived from the work by Langella et al. They showed that the PG domain belongs to the family of IDPs, mainly being unfolded with only local tendencies in the N-terminus to assume PPII conformations [31,34]. As the PPII are reported to play a role in several cellular processes, including partner recognition and possibly cell adhesion and intercellular communications [35], the PG domain could play a similar role. SAXS data analysis and modeling sheds light on the inherent disorder of the PG domain and shows that the PG domain can probably populate a number of different “average” positions and conformations. This inherent disorder, shown for the first time in the full ECD of CA IX, leaves all above open, but can explain our inability to obtain a crystal structure of the full CA IX ECD.

It is known that the ECD of CA IX is shed in cancer patients and there are several commercial kits that allow for the detection of CA IX ECD shedding from patient serum or plasma [70]. The capturing antibody (V10) recognizes the CA domain, while the other detection antibody (M75) binds to the PG domain, thereby only showing full ECD domain. Studies by Zatovicova and colleagues show that both the CA domain and PG domain are present in serum and plasma circulating ECD of CA IX [71]. To the best of our knowledge, a spontaneous separation of the PG and catalytic domain of CA IX has not been reported in vivo. The use of commercial assays and the order in which the available antibodies are used to detect CA IX does not allow for the PG and catalytic domain to be observed separately. Based on the observation of the loss of the PG domain in vitro, it is tempting to speculate whether there is a similar physiological process where there is additional processing of circulating or even membrane-bound CA IX to remove the PG domain only. If so, what would the purpose be? Investigation of the ECD sequence against known proteases reveal neutrophil elastase is the only one that produces peptides consistent with our MS data. Neutrophil elastase is a powerful serine protease that can also be found in the extracellular matrix and is responsible for digestion of a number of proteins [72,73]. In this setting, it would also be able to process the ECD of CA IX to produce freely circulating CA IX and/or PG domain only. Our results suggest that the PG domain occupies preferred conformations around the CA domain, and it could be that in some conformations, it is more accessible to a protease. Cleavage (shedding) of the PG domain only, independent of shedding the whole ECD, could be enabled when in different conformations. Even though the physiological relevance is elusive, these observations might open up a new research question as to whether the dynamics and potential shedding of the PG domain has an undiscovered role in cancer progression.

## Figures and Tables

**Table 1:** Crystallographic data collection and refinement statistics.

Data Collection and Processing	PDB ID 6y74
Source	BioMAX (MAX IV Laboratory)
^1^ Wavelength (Å)	0.979
Detector	DECTRIS, Eiger 16M Hybrid-pixel detector
^2^ Rotation range per image (°)	0.1
Total number of images	3600
Space group, unit cell (Å, °)	C2, *a* = 113.9, *b* =78.3, *c* = 74.3; β = 128.2
Resolution range (Å)	37.13–1.53 (1.56–1.53)
Total No. of reflections	519,006 (21,203)
No. of unique reflections	75,610 (3288)
Multiplicity	6.9 (6.4)
Completeness (%)	85.0 (98.3)
I/σ(I)	10.6 (2.2)
^3^ R-merge (%)	0.110 (0.815)
CC(1/2)	0.76 (0.99)
Wilson B	15.6
^4^ R_cryst_/R_free_ (%)	17.3/19.8
Rmsd bond lengths, angles (Å, °)	0.016/1.723
Ramachandran favored/outliers/rotamer outliers %	97.1/0.0/1.34
Model clash score	6.31
No. solvent molecules	606
Mean B factor protein/solvent/	20.2/29.0
Mean B factor Zn/sugar/acetate	13.3/44.1/24.7

^1^ Å = Ångstrom, ^2^ ° = degrees, ^3^ R_merge_ = ∑*_hkl_* ∑*_i_* |*I_i_*(*hkl*)−〈*I*(*hkl*)〉 |/∑*_hkl_* ∑*_i_ I_i_*(*hkl*), where *I_i_*(*hkl*) is the observed intensity and *I*(*hkl*) is the average intensity over symmetry equivalents. ^4^ R_cryst_ = ∑*_hkl_* ||*F_obs_*|−|*F_calc_*| |/∑*_hkl_* |*F_obs_*|. R_free_ was calculated in the same way, but from a randomly chosen 5% of reflections which were omitted from the refinement process.

## Supplementary Materials

Supplementary materials can be found at https://www.mdpi.com/1422-0067/21/15/5277/s1.
