# Supplementary material for: Biophysical Characterization of Cancer-Related Carbonic Anhydrase IX
Source: Int J Mol Sci. 2020 Jul 25;21(15):5277. doi: 10.3390/ijms21155277 (PMC7432807; doi:10.3390/ijms21155277)
Supplement: Supplementary file 1 [file ijms-21-05277-s001.pdf]

SUPPORTING MATERIAL

Table S1. Essential SAS data acquisition and analysis information, based on the template from Trewhella et al. [1].

**a) Sample details**	
**Partial specific volume $\bar{\nu}$ from chemical composition (cm^{3}g^{-1})**	0.728
Particle contrast from sequence and solvent constituents, $\Delta\bar{\rho}$ ($\rho_{protein} - \rho_{solvent}$; 10^{10} cm^{-2})	2.951 (12.427-9.475)
Mass from chemical composition (Da)	40913
SEC-SAXS	
Column	Superdex 200 5/150
Loaded concentration (mg ml^{-1})	1.5
Injection volume (μL)	150
Flow rate (ml min^{-1})	0.3
Solvent (solvent blanks taken from SEC flowthrough prior to elution of protein)	50 mM Tris pH 7.5 and 150 mM NaCl
**b) SAXS data-collection parameters**	
Instrument	MacCHESS BioSAXS beamline G1 at CHESS (now ID7A1) with Dectris Pilatus 100k detector
Wavelength (Å)	1.245
Beam size (μm^2)	250 x 250
Camera length (m)	1.4982
q measurement range (Å^{-1})	0.0087-0.283
Absolute scaling method	N/A
Normalization	To transmitted intensity by beam-stop counter
Monitoring for radiation damage	Frame-by-frame comparison of data
Exposure time	Continuous 1 s exposures during SEC elution
Sample configuration	Custom made flat-sided sample cell with polystyrene windows [2].
Sample temperature (ºC)	20
**c) Software employed for SAXS data reduction, analysis, and interpretation**	
SAXS data reduction	Radial averaging using BioXTAS RAW 0.99.14b [3], frame comparison, averaging, and subtraction done using BioXTAS RAW 1.6.3 [4].
Extinction coefficient used	5.2 X 10^4 M^{-1} cm^{-1}
Calculation of $\Delta\bar{\rho}$ and $\bar{\nu}$	MULCh 1.1.1 (20/01/23; [5])
Basic analysis: Guinier, MW, Normalized Kratky, P(r)	RAW 1.6.3, P(r) function using GNOM [6] from ATSAS 2.8.4. RAW uses V_c and MoW2 molecular weight methods [7, 8].
Atomic structure modeling	EOM 2.0 from ATSAS 2.8.4 [9].
**d) Structural parameters**	
Guinier analysis	
I(0) (Arb.)	2.90 ± 0.06
R_g (Å)	25.2 ± 0.9
q-range (Å^{-1})	0.00869-0.05156
$q_{max}R_g$	1.298
Coefficient of correlation, r^2	0.637
Volume (Å^3, adjusted V_P as SAXS MoW2)	38923
MW, MoW2 method (kDa) (ratio to expected)	32.3
MW, Vc method (kDa) (ratio to expected)	36.1

P(r) analysis	
I(0) (Arb.)	2.90 ± 0.06
R_g (Å)	26.2 ± 0.7
D_{max} (Å)	85
q-range ($Å^{-1}$)	0.00869-0.283
χ^2	1.19
Total estimate	0.945
**e) Shape model-fitting results**	
	CA IX with PG domain
DAMMIF (default parameters, 15 models, slow mode)	
q range for fitting ($Å^{-1}$)	0.00869-0.283
Symmetry, anisotropy assumptions	P1, none
NSD (standard deviation), number of clusters.	0.995 (0.079), 2
χ^2 range	1.193
Constant adjustment to intensities	0.0376
DAMMIN refinement (default parameters)	
q range for fitting ($Å^{-1}$)	0.00869-0.283
Symmetry, anisotropy assumptions	P1, none
χ^2	1.193
Constant adjustment to intensities	0.03306
M estimate (kDa)	49.64
**f) Atomistic modelling**	
	CA IX with PG domain
Crystal structure	PDB 3IAI
q-range for fitting ($Å^{-1}$)	0.00869-0.283
Symmetry assumptions	None
χ^2	1.189
Constant subtraction	0.031
No. of representative structures	3
Ensemble (pool) average R_g (Å)	25.89 (32.14)
Ensemble (pool) average D_{max} (Å)	87.03 (115.63)
Ensemble (pool) average volume ($Å^3$)	59429.64 (66002.52)
Ensemble (pool) average Ca(N)-Ca(C) distance (Å)	56.05 (88.42)
R_{flex} ensemble (pool)	52.65 % (83.33 %)
R_{sigma} ensemble (pool)	1.66

Table S2. Interactions at the interface of the monomer in the non-crystallographic dimer.

**monomer A**	**monomer B**	**Type of interaction**
Leu206 (O)	Arg261 (s.c.)	H-bond
Arg221 (s.c.)	Thr257 (O)	H-bond
Leu223 (O)	Arg261 (s.c.)	H-bond
Thr257 (O)	Arg221 (s.c.)	H-bond
Arg261 (s.c.)	Leu206 (O)	H-bond
Ala258 (O)	Gly209 (N)	Solvent-mediated (A660)
Ala260 (O)	Val262 (N)	Solvent-mediated (A680)
Glu264 (s.c.)	Leu206 (O)	Solvent-mediated (A602)
Leu255 (s.c.), Leu223 (s.c.)	Ala260 (s.c.)	Hydrophobic

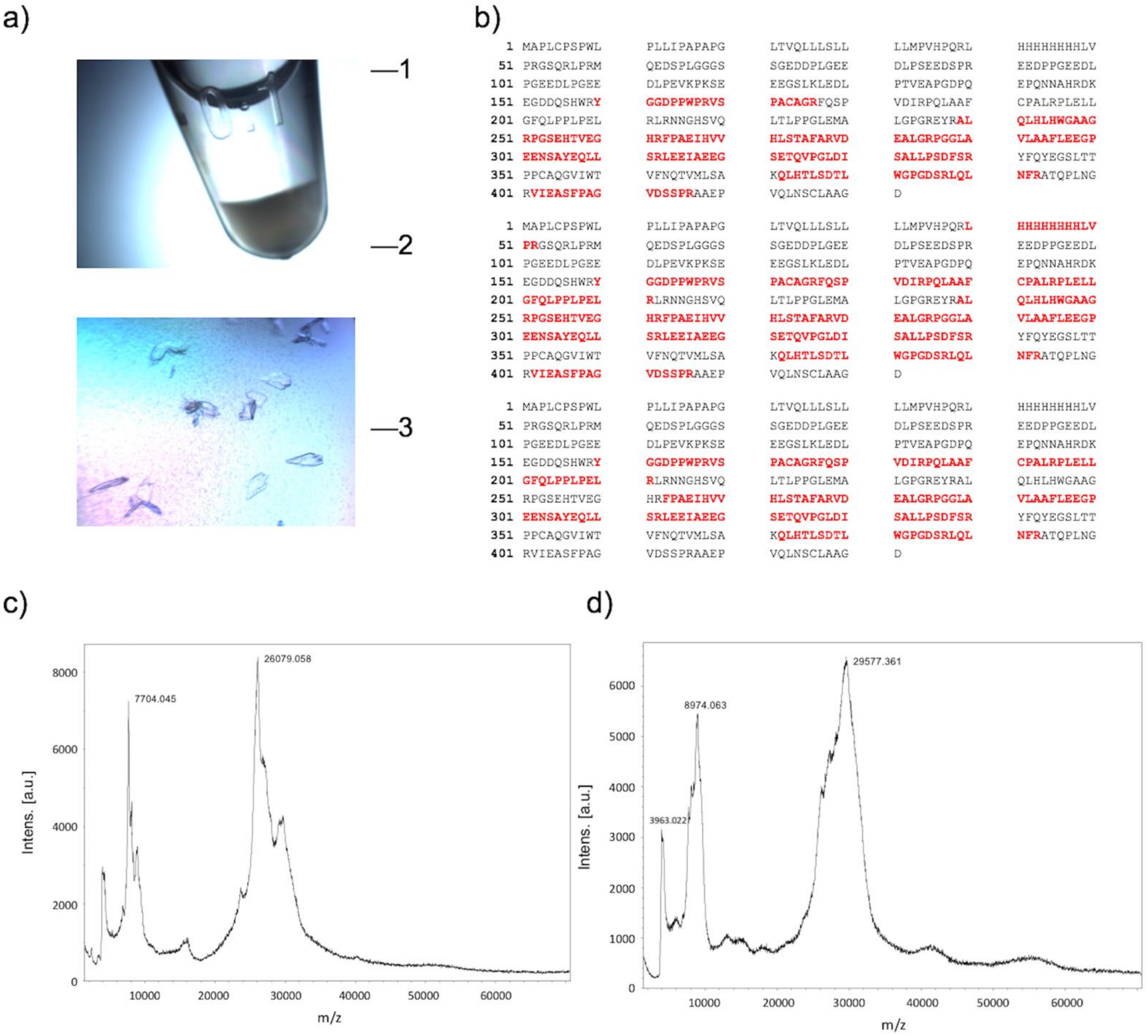

Figure S1. Peptide or protein masses identified from intact and trypsin-digested samples using MALDI-TOF mass spectrometry. (a) This insert shows the different samples that were analyzed using mass spectrometry: 1) soluble component of CA IX used for crystallization, 2) insoluble component of CA IX that appeared after long-term storage, 3) crystals of CA IX that were crushed for mass spectrometry analysis; (b) Peptides identified using MALDI-TOF MS of trypsin-digested samples shown in the same order as laid out in (a). The identified peptides are highlighted in red within the full-length sequence of CA IX; (c) and (d) show the intact mass measurements for soluble CA IX component (a1) and the washed and crushed crystals (a3), respectively. As the peaks are noisy and broad the mass was estimated at ~28 kDa using full-width at half the maximal height of the peaks gives ~ 28 kDa, consistent with the calculated mass from the crystal structure and migration on SDS-PAGE.

(a)

Start	–	End	Observed	Mr(expt)	Mr(calc)	Peptide
160	–	168	1044.48	1043.47	1043.4825	R.YGGDPPWPR.V
169	–	176	760.37	759.36	759.3698	R.VSPACAGR.F
239	–	262	2616.30	2615.29	2615.3218	R.ALQLHLHWGAAGRPGSEHTVEGHR.F
263	–	278	1794.95	1793.94	1793.9577	R.FPAEIHVVHLSTAFAR.V
279	–	312	3600.82	3599.82	3599.8107	R.VDEALGRPGGLAVLAAFLEEGPEENSAYEQLLSR.L
313	–	340	3002.46	3001.45	3001.4768	R.LEEIAEEGSETQVPGLDISALLPSDFSR.Y
372	–	387	1782.86	1781.85	1781.8697	K.QLHTLSDTLWGPGDSR.L
388	–	393	790.44	789.43	789.4497	R.LQLNFR.A
402	–	416	1531.76	1530.75	1530.7678	R.VIEASFPAGVDSSPR.A

(b)

Start	–	End	Observed	Mr(expt)	Mr(calc)	Peptide
40	–	52	1693.83	1692.82	1692.8723	R.LHHHHHHHHLVPR.G
160	–	168	1044.47	1043.46	1043.4825	R.YGGDPPWPR.V
169	–	176	760.36	759.35	759.3698	R.VSPACAGR.F
177	–	211	3942.13	3941.12	3941.1753	R.FQSPVDIRPQLAAFCPALRPLELLGFQLPPLPELR.L
239	–	262	2616.26	2615.26	2615.3218	R.ALQLHLHWGAAGRPGSEHTVEGHR.F
263	–	278	1794.92	1793.91	1793.9577	R.FPAEIHVVHLSTAFAR.V
279	–	312	3600.76	3599.75	3599.8107	R.VDEALGRPGGLAVLAAFLEEGPEENSAYEQLLSR.L
313	–	340	3002.41	3001.40	3001.4768	R.LEEIAEEGSETQVPGLDISALLPSDFSR.Y
372	–	387	1782.83	1781.82	1781.8697	K.QLHTLSDTLWGPGDSR.L
388	–	393	790.42	789.41	789.4497	R.LQLNFR.A
402	–	416	1531.74	1530.73	1530.7678	R.VIEASFPAGVDSSPR.A

(c)

Start	–	End	Observed	Mr(expt)	Mr(calc)	Peptide
160	–	168	1044.52	1043.51	1043.4825	R.YGGDPPWPR.V
169	–	176	760.38	759.38	759.3698	R.VSPACAGR.F
177	–	211	3942.29	3941.29	3941.1753	R.FQSPVDIRPQLAAFCPALRPLELLGFQLPPLPELR.L
263	–	278	1794.99	1793.98	1793.9577	R.FPAEIHVVHLSTAFAR.V
279	–	312	3600.85	3599.85	3599.8107	R.VDEALGRPGGLAVLAAFLEEGPEENSAYEQLLSR.L
313	–	340	3002.47	3001.46	3001.4768	R.LEEIAEEGSETQVPGLDISALLPSDFSR.Y
372	–	387	1782.90	1781.89	1781.8697	K.QLHTLSDTLWGPGDSR.L
388	–	393	790.47	789.46	789.4497	R.LQLNFR.A

Figure S2. Peptide masses identified from trypsin-digested samples using MALDI-TOF MS. (a) soluble component of CA IX used for crystallization, (b) insoluble component of CA IX that appeared after long-term storage, (c) crystals of CA IX that were crushed for mass spectrometry analysis.

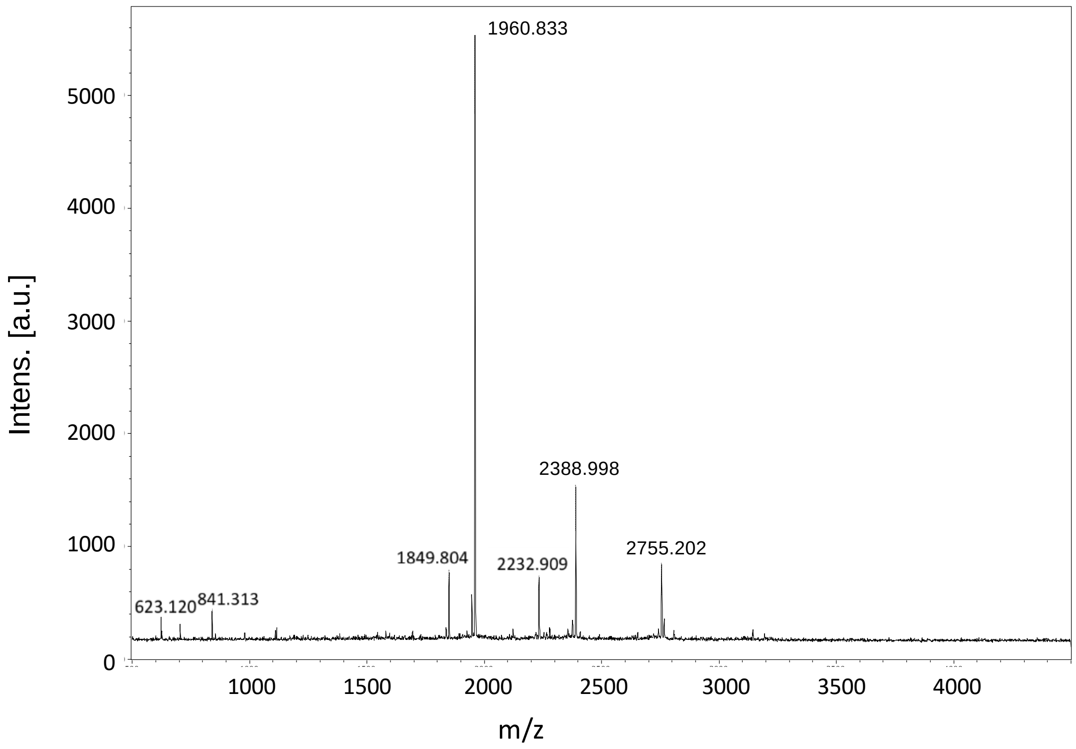

Figure S3. Mass spectra of the insoluble component showing one major peak at MW 1960 Da. This peak corresponds to peptide sequence HPQRLHHHHHHHHLV and could only be generated by neutrophil elastase.

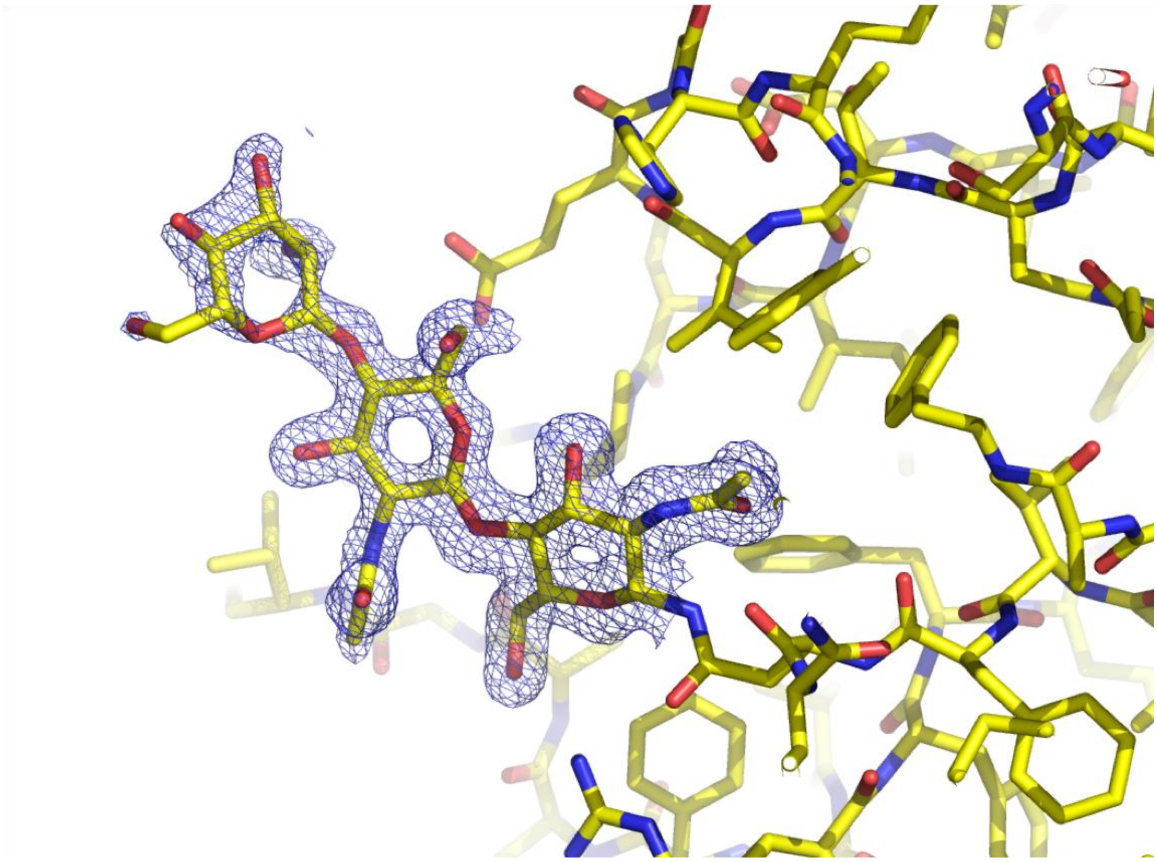

Figure S4. Glycosylation site of CA IX shown as yellow sticks. The 2Fo-Fc electron density map is shown in blue mesh at 1.5σ level for the sugars only.

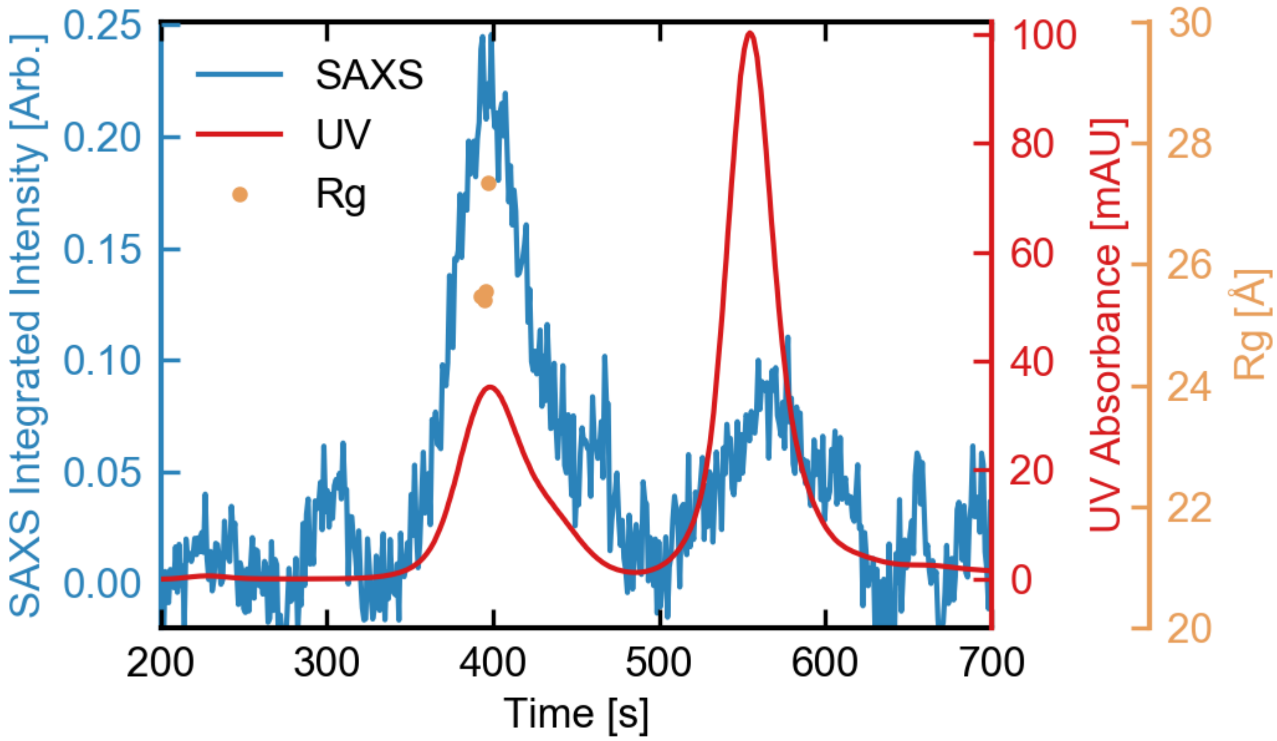

Figure S5. The total scattering intensity as a function of time from SEC-SAXS (a 'scattergram', with peaks where protein elutes), blue, is plotted on the left axis. The corresponding UV absorbance at 280 nm (red) is plotted on the right axis. The automatically calculated R_g for each time point (orange) is plotted on the second right axis. Due to the low signal to noise of the data, only a few points have successfully calculated R_g values, so the data required further treatment. The UV chromatogram and the SAXS scattergram have been aligned in time by applying an arbitrary time offset to the UV chromatogram.

References

1. Trewhella, J.A.P.; Duff, D.; Durand, F.; Gabel, J.M.; Guss, W.A.; Hendrickson, G.L.; Hura, D.A.; Jacques, N.M.; Kirby, A.H. Kwan, J. et al. 2017 publication guidelines for structural modelling of small-angle scattering data from biomolecules in solution: an update. *Acta Crystallogr. Sect. D. Biol. Crystallogr.*, **2017**, *73*, 710–728.
2. Acerbo, A.S.; Cook, M.J.; Gillilan, R.E. Upgrade of MacCHESS facility for X-ray scattering of biological macromolecules in solution. *J. Synchrotron Radiat*. **2015**, *22*, 180–186.
3. Nielsen, S.S.; Toft, K.N.; Snakenborg, D.; Jepp.esen, M.G.; Jacobsen, J.K.; Vestergaard, B.; Kutter, J.P.; Arleth, L. BioXTAS RAW, a software program for high-throughput automated small-angle X-ray scattering data reduction and preliminary analysis. *J. Appl. Crystallogr*. **2009**, *42*, 959–964.
4. Hopkins, J.B.; Gillilan, R.E.; Skou, S. BioXTAS RAW: Improvements to a free open-source program for small-angle X-ray scattering data reduction and analysis. *J. Appl. Crystallogr*. **2017**, *50*, 1545–1553.
5. Whitten, A.E.; Cai, S.; Trewhella, J. MULCh: Modules for the analysis of small-angle neutron contrast variation data from biomolecular assemblies. *J. Appl. Crystallogr*. **2008**, *41*, 222–226.
6. Svergun, D.I. Determination of the regularization parameter in indirect-transform methods using perceptual criteria. *J. Appl. Crystallogr*. **1992**, *25*, 495–503.
7. Piiadov, V.; Ares de Araújo, E.; Oliveira Neto, M.; Craievich, A.F.; Polikarpov, I. SAXSMoW 2.0: Online calculator of the molecular weight of proteins in dilute solution from experimental SAXS data measured on a relative scale. *Protein Sci*. **2019**, *28*, 454–463.

8. Rambo, R.P.; Tainer, J.A. Accurate assessment of mass, models and resolution by small-angle scattering. *Nature* **2013**, *496*, 477–481.
9. Tria, G.; Mertens, H.D.; Kachala, M.; Svergun, D.I. Advanced ensemble modelling of flexible macromolecules using X-ray solution scattering. *IUCrJ* **2015**, *2*, 207–217.
